# LnCRNAs in the Regulation of Endometrial Receptivity for Embryo Implantation

**DOI:** 10.5935/1518-0557.20240038

**Published:** 2024

**Authors:** Haniyeh Ebrahimnejad Hasanabadi, Azam Govahi, Shahla Chaichian, Mehdi Mehdizadeh, Ladan Haghighi, Marziyeh Ajdary

**Affiliations:** 1 Department of Pediatric Nursing and NICU, School of Nursing & Midwifery, Tehran University of Medical Sciences, Tehran, Iran; 2 Endometriosis Research Center, Iran University of Medical Sciences, Tehran, Iran; 3 Reproductive Sciences and Technology Research Center, Department of Anatomy, Iran University of Medical Sciences, Tehran, Iran

**Keywords:** embryo implantation, endometrial receptivity, endometrium, lncRNA

## Abstract

The development of endometrial receptivity is crucial for successful embryo implantation and the initiation of pregnancy. Understanding the molecular regulatory processes that transform the endometrium into a receptive phase is essential for enhancing implantation rates in fertility treatments, such as in vitro fertilization (IVF). Long non-coding RNAs (lncRNAs) play a pivotal role as gene regulators and have been examined in the endometrium. This review offers current insights into the role of lncRNAs in regulating endometrial receptivity. Considering the significant variation in endometrial remodeling among species, we summarize the key events in the human endometrial cycle and discuss the identified lncRNAs in both humans and other species, which may play a crucial role in establishing receptivity. Notably, there are 742 lncRNAs in humans and 4438 lncRNAs that have the potential to modulate endometrial receptivity. Additionally, lncRNAs regulating matrix metalloproteinases *(MMPs)* and Let-7 have been observed in both species. Future investigations should explore the potential of lncRNAs as therapeutic targets and/or biomarkers for diagnosing and improving endometrial receptivity in human fertility therapy.

## INTRODUCTION

Effective pregnancy implantation relies on a complicated molecular cross-talk between the mother’s uterus and the developing conceptus. In pigs, maternal and fetal contact occurs approximately three times on the 12^th^ day of pregnancy. Implantation in pigs is associated with the dynamic production of estrogen, progesterone, prostaglandins, adhesion molecules, and immune factors. To achieve successful implantation, suitable endometrium, embryo quality, and molecular cellular changes in the uterine environment are required. The most crucial factor is the receptive endometrium, which undergoes significant cellular and molecular changes from non-receptive to receptive. If the endometrium is not receptive, the blastocyst cannot be implanted (Evans *et al.,* 2016).

The human endometrium as a dynamic tissue experiences regular regeneration during the menstrual cycle, and the uterus is receptive to embryo implantation for only a short period (Gellersen *et al.,* 2007; Evans *et al.,* 2016). It has been estimated that 1.3% of implantation failures in healthy women result from the non-acceptance of endo-metrial tissue (Altmäe *et al.,* 2017). In patients undergoing in vitro fertilization (IVF) cycles, 60 to 70% of patients with high embryo quality cannot implant successfully due to non-receptive endometrium (Paulson *et al.,* 1997; Heng *et al.,* 2011). Therefore, awareness of the endometrial receptivity molecular regulation is essential in increasing implantation rates and fertility therapy.

Endometrial remodeling varies between human species (menstrual cycle) and animals (estrous cycle) (Johnson, 2018; Shekibi *et al.,* 2022). It is also known that changes in the cellular and molecular levels of the uterine environment can affect endometrial receptivity (Salamonsen *et al.,* 2009; Lessey & Young, 2019; Ochoa-Bernal & Fazleabas, 2020).

Biochemically, invasion mechanisms involved in embryo implantation include apoptosis and other mechanisms for epithelial breakdown, cell-cell or cell-substrate interactions that contribute to migration, vascular and extracellular matrix (ECM) remodeling, as well as immune responses involving adaptive and innate immune cells (Hernández-Vargas *et al.,* 2020). Despite advances in assisted reproductive technology (ART), knowledge about embryo implantation remains incomplete. The cellular and molecular changes in endometrial tissue are the most important factors in the success or failure of embryo implantation. Identifying biomarkers to improve the chances of pregnancy in ART cycles is crucial. Although studies on small molecules have shed some light on the mechanism of embryo implantation failure, there has not been an improvement in the pregnancy rates and implantation of clinical embryos. Thus, new methods are essential to enhance embryo implantation efficiency.

Long non-coding RNAs (lncRNAs) as RNA transcripts have more than 200 nucleotides with no or little protein-coding capacity without an effective open reading frame. They have been considered in the past few years and have functional roles in chromatin modification, epigenetic regulation, transcriptional control, genomic imprinting, and pre- and post-translational mRNA processing. LncRNAs similar to mRNAs can be transcribed by spliced, RNA polymerase II, polyadenylated, and presumably capped at the 5’ ends (Li *et al.,* 2019).

In this review, we discuss the lncRNAs that are effective in endometrial receptivity and their potential use as therapeutic target biomarkers in human fertility treatment. Abnormalities in lncRNA profiles can significantly affect the implantation ability of uterine tissues, which can be one of the important causes of endometrial receptivity disorder.

## A REVIEW OF THE ENDOMETRIAL CYCLE IN HUMANS

### Endometrial Receptivity and Human Menstrual Cycle

#### Proliferative Phase

The first stage of the menstrual cycle is the follicular or proliferative phase. It occurs from the first day to the 14^th^ day of the menstrual cycle, based on an average duration of 28 days. Changes in the length of the menstrual cycle occur due to changes in the length of the follicular phase. The main hormone in this phase is estrogen, especially 17-beta estradiol. The increase of this hormone occurs by regulating the FSH receptors in the follicle at the beginning of the cycle. However, as the follicular phase progresses to the end, increased amounts of 17-beta-estradiol provide negative feedback to the anterior pituitary. The purpose of this stage is the growth of the endometrial layer of the uterus. 17-beta-estradiol achieves this by increasing the growth of the endometrial layer of the uterus, stimulating increased amounts of stroma and glands, and increasing the depth of the arteries that supply the endometrium, the spiral arteries. 17-Beta-estradiol achieves this by creating channels in the cervix, allowing sperm to enter (Herbison, 2020). During this stage, a primordial follicle begins to mature into a Graafian follicle. The surrounding follicles begin to degenerate, this is when the Graafian follicle becomes the mature follicle. This sets the follicle up for ovulation (Stankewicz *et al.,* 2024).

#### Ovulation

Ovulation always occurs 14 days before menstruation. Therefore, with an average cycle of 28 days, ovulation occurs on day 14. At the end of the proliferative phase, the level of 17-beta-estradiol is at a high level due to follicle maturation and increased hormone production. Only during this time, 17-beta-estradiol provides positive feedback for the production of FSH and LH. This occurs when a critical level of 17-beta-estradiol is reached, i.e. at least 200 pg/ml of plasma. The high levels of FSH and LH present at this time is called the LH surge. As a result, the mature follicle ruptures, and an egg is released (Stankewicz *et al.,* 2024).

#### Secretory Phase

**Figure 1:**
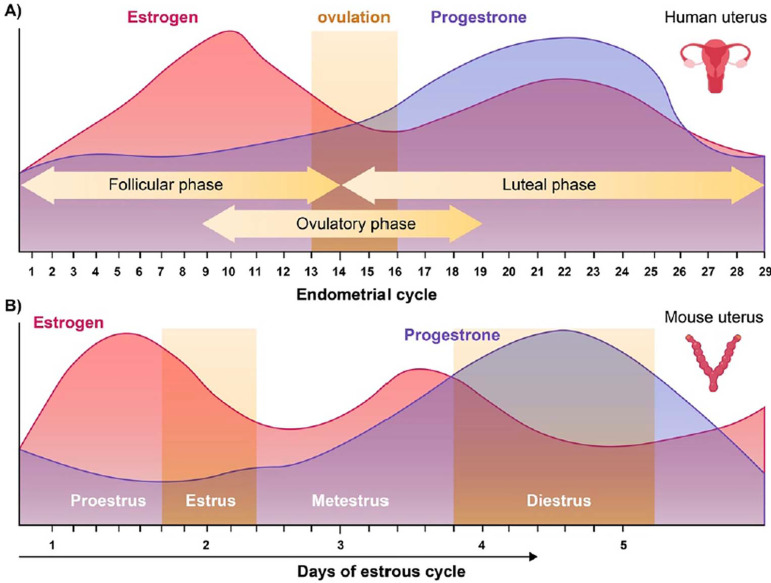
(A) Endometrial changes across the menstrual cycle in humans. (B) Endometrial changes across the Estrus cycle.

This phase always occurs from day 14 to day 28 of the cycle. Progesterone stimulated by LH is the dominant hormone at this stage to prepare the corpus luteum and endometrium for the implantation of a possible fertilized egg. As the luteal phase ends, progesterone provides negative feedback to the anterior pituitary to decrease FSH and LH levels and subsequently 17-beta-estradiol and progesterone levels. The corpus luteum is a structure formed in the ovary at the site of mature follicle rupture and produces 17 beta-estradiol and progesterone, which is dominant at the end of the phase due to the negative feedback system. The endometrium is prepared by increasing vascular reserve and stimulating mucous secretions. This is achieved by stimulation of the endometrium by progesterone to slow down the proliferation of the endometrium, reduce the thickness of the lining, develop more complex glands, accumulate energy sources in the form of glycogen, and create more surface area inside the spiral arteries. Near the end of the secretory phase, plasma levels of 17-beta-estradiol and progesterone are produced by the corpus luteum (Bajpai *et al.,* 2023) (Figure 1).

#### Normal Menstruation

When the hormone level decreases, the endometrial layer, as it has changed during the menstrual cycle, is not able to maintain. This is called menstruation, which is considered to be days 0 to 5 of the next menstrual cycle. Menstrual blood is mainly arterial and only 25% of blood is venous. It contains prostaglandins, tissue debris, and relatively large amounts of fibrinolysis from endometrial tissue. The normal duration of menstruation is 3 to 5 days, but menstruation as long as 1 day and up to 8 days can occur in a normal woman (Bajpai *et al.,* 2023).

### LnCRNAs and Endometrial Receptivity

Non-coding RNAs are one of the epigenetic modifications that regulate endometrial decidualization. LncRNAs exhibit very low species conservation amongst various species with high specificity in various cells and tissues (Li & Chang, 2014). They are involved in normal biological processes as well as the development and occurrence of complex illnesses (Bartonicek *et al.,* 2016; Schmitz *et al.,* 2016).

The regulation of lncRNAs in human endometrial decidualization has recently been considered. Earlier studies revealed significant expression of lncRNAs in hESCs from patients with diverse diseases (Monnier *et al.,* 2013; Ghazal *et al.,* 2015; Fan *et al.,* 2017; Wang *et al.,* 2018; Chen *et al.,* 2019). Subsequently, relevant studies have delved into the regulatory mechanisms of lncRNAs during endometrial decidualization (Liang *et al.,* 2016; Zhao *et al.,* 2022a). However, the regulatory mechanism of ln-cRNAs in the pathological state’s abnormal decidualization of the endometrium remains unclear. lncRNAs participate in regulating various biological processes, including carcinogenesis, epigenetic regulation, and embryonic development (Wapinski & Chang, 2011; Subramanian *et al.,* 2013). It is attempted to identify important lncRNAs as biomarkers to anticipate endometrial receptivity (Koot *et al.,* 2016; Sigurgeirsson *et al.,* 2017; Vireque *et al.,* 2017; Feng *et al.,* 2018; Chen *et al.,* 2019). lncRNA expression has been characterized in the female reproductive tract, particularly during the peri-implantation period when expressed in the uterus.

### Identification of LnCRNAs involved in implaπtation

One of the essential criteria for successful implantation is the quality of the endometrial tissue and its epithelial and stromal cells, which are in direct contact with the blastocyst. Decidualization should occur efficiently for implantation to progress. The role of lncRNAs in humans in implantation progress has been studied. The endometrium can also be affected by embryo implantation (Zhao *et al.,* 2022a;b). Due to the increasing application of IVF methods, spent blastocyst cultures have been analyzed for lncRNA and other biomarkers (Azevedo-Quintanilha *et al.,* 2019).

While previous reviews on lncRNAs and embryos often discussed embryo development as well as implantation, some studies have noted that lncRNAs secreted by pre-implantation blastocysts can facilitate embryo-endometrial communication to improve implantation. However, we did not explain this issue here in detail. Embryo-derived ln-cRNAs also play a role in implantation failure.

Unlike mRNA, which acts based on RNA strand modifications to increase its stability (Azevedo-Quintanilha *et al.,* 2019), lncRNAs are not directly translated into protein (Spizzo *et al.,* 2012; Tahermanesh *et al.,* 2023). Many studies have shown that lncRNAs play vital roles in several biological processes using mechanisms, like genetic imprinting, mRNA degradation, chromatin remodeling, mRNA editing, splicing regulation, and translation regulation (Geisler & Coller, 2013; Zhu *et al.,* 2013). Examples of such lncRNAs are shown in Table 1.

**Table 1:** Identification of LnCRNAs involved in implantation.

Target	L∏CRNA	Species	Target Gene/Protein	Improved/Impaired Receptivity	Ref
endometrial tissues	*TCONS_0Ί729386*	Human	*FGF7, NMB, FGF9, VEGFC, VEGFA, Mucl, ESR1 and RBP4*↑	Improved receptivity	(Wang *et al.,* 2016)
endometrial tissues	*TCONS_01325501*	Human	*FGF7, NMB, FGF9, VEGFC, VEGFA, Mucl, ESR1 and RBP4*↑	Improved receptivity	(Wang *et al.,* 2016)
endometrial tissues	*LncRNA-TCL6*	Human	*EGFR, ERK, and AKT*↓	Impaired Receptivity	(Liu & Gong, 2018)
Endometrial tissue	*H19*	Human	↓*IGFBP1*	Impaired Receptivity	(Ariel *et al.,* 1997; Adriaenssens *et al.,* 1999; Kallen *et al.,* 2013)
Endometrial tissue	*CCDC144NL-AS1*	Human	↓*MMP9*	Impaired Receptivity	(Zhang *et al.* 2018)
Endometrial tissue	IncRNA H19	Human	↑*Let 7*, ↓ ITGB3	Impaired Receptivity	(Zeng *et al.* 2017)
Epithelial Cell	*IncRNA TCL1*	Human	*TUNAR*↑	Improved receptivity	(Wang et *al.* 2020b)
Epithelial Cell	*IncRNA FTX*	Human	*E-cadherin*↑, ↓*vimentin, N-cadherin, and zinc finger E-bo× binding homeobo× 1*	Improved receptivity	(Wang *et al.,* 2020a)
Stroma cell	*IncSAMD11-1:1*	Human	↓ *PIP4K2A*	Impaired Receptivity	(Zhang *et al.,* 2022)
Stroma cell	*IncRNA TCL1*	Human	*TUNAR*↑	Improved receptivity	(Wang *et al.,* 2020b)
Stroma cell	*NONHSAT083203.2*	Human	*CECR3*↑	Impaired Receptivity	(Feng *et al.,* 2018)
Stroma cell	*NONHSAT212577.1*	Human	*ST7-OT3*↑	Impaired Receptivity	(Feng *et al.,* 2018)
Stroma cell	*NONHSAT035952.2*	Human	*DHRS4-AS1*↑	Impaired Receptivity	(Feng *et al.,* 2018)
Stroma cell	*NONHSAT193031.1*	Human	*C22orf34*↑	Impaired Receptivity	(Feng *et al.,* 2018)
Stroma cell	*NONHSAT053761.2*	Human	*RAMP2-AS1*↑	Impaired Receptivity	(Feng *et al.,* 2018)
Stroma cell	*NONHSAT025064.2*	Human	*PNCT_HSA157732*↑	Impaired Receptivity	(Feng *et al.,* 2018)
Stroma cell	*IncRNA FTX*	Human	*E-cadherin*↑, ↓*vimentin, N-cadherin, and zinc finger E-bo× binding homeobo× 1*	Improved receptivity	(Wang *et al.,* 2020a)
Stroma cell	*LINC473*	Human	↑*PRL, IGFBP1, PGR, FOXOl, HOXA10*	Impaired Receptivity	(Liang *et al.,* 2016)
Stroma cell	*HOXA11 antisense*	Human	↓*HOXA11*	Impaired Receptivity	(Chau *et al.,* 2002)
Stroma cell	*PTENP1*	Human	↑miR-590-3p	Impaired Receptivity	(Takamura *et al.,* 2020)
Serum	*aHIF*	Human	*VEGF*↑	Impaired Receptivity	(Qiu *et al.,* 2019)

In human endometrial tissue, the expression of *TCONS_01729386* increases the expression of Fibroblast Growth Factor 7 *(FGF7),* Neuromedin B *(NMB),* fibroblast growth factor-9 *(FGF9),* Vascular Endothelial Growth Factor C (VEGFC), Vascular Endothelial Growth Factor A *(VEGFA),* Mucin 1 *(Muc1),* Estrogen Receptor 1 *(ESR1),* and Retinol Binding Protein 4 *(RBP4)* genes (Wang *et al.,* 2016), while *TCONS_01325501* also increases the expression of these genes (Wang *et al.,* 2016). According to microarray studies, it has been proven that when the expression of these genes increases, the rate of implantation increases (Herington *et al.,* 2016). These genes help to improve implantation by increasing the rate of angiogenesis, proliferation, and invasion, and reducing apoptosis (Herington *et al.,* 2016).

Additionally, gi|672027621 decreases Pyrimidinergic Receptor P2Y6 (*P2ry6*) expression, while *gi*|*672045999* reduces A disintegrin and metalloproteinase with thrombo-spondin motifs 7 *(Adamts7*) expression and improves implantation (Cai *et al.,* 2019). Furthermore, in endometrial tissue, the expression of *lncRNA-* matrix metalloproteinase-11 *(MMP11)* increases the expression of *MMP11* (Zhao *et al.,* 2022a;b;c), and *lncRNA-TCL6* decreases *Epidermal growth factor receptor (EGFR),* extracellular signal-regulated kinases (*ERK*), and *AKT* gene expression in human endometrial tissue (Liu & Gong, 2018). These genes help to improve implantation by increasing the rate of proliferation, and invasion and reducing apoptosis (Herington *et al.,* 2016).

*CCDC144NL-AS1* decreases *MMP9* expression in endometrial tissue (Zhang *et al.,* 2018), while H19, by affecting lethal-7 *(Let* 7), decreases Integrin alpha-IIb/beta-3 *(ITGB3)* gene expression and reduces the implantation rate in human endometrial tissue (Zeng *et al.,* 2017). *ITGB3* helps to improve implantation by increasing the rate of angiogenesis (Herington *et al.,* 2016).

In epithelial cells of human endometrial tissue, the expression of *lncRNA T-Cell Leukemia/Lymphoma 1* (*TCL1*) and five prime to Xist (*FTX*) increases the expression of *TUNAR* and *E-cadherin,* respectively, while decreasing the expression of *N-cadherin, vimentin,* and zinc finger E-box binding homeobox 1, thereby improving implantation (Wang *et al.,* 2020a;b). Meanwhile, in human epithelial cells, the expression of *PTENP1* increases the expression of *miR-590-3p* and destroys implantation (Takamura *et al.,* 2020), These genes help to improve implantation by increasing the cell mobility and decidualization in cell endometrium (Wu *et al.,* 2023).

In human stromal cells, the expression of *lncRNA TCL1* increases TCL1 Upstream Neural Differentiation-Associated RNA *(TUNAR)* expression (Wang *et al.,* 2020b), while *lncRNA FTX* increases the expression of E-cadherin and decreases the N-cadherin, zinc finger E-box binding homeobox 1 and vimentin expression, thereby improving implantation (Wang *et al.,* 2020a). On the other hand, *lncSAMD11-1:1* downregulates Phosphatidylinositol-5-Phosphate 4-Kinase Type 2 Alpha (*PIP4K2A*) expression (Zhang *et al.,* 2022), and *NONHSAT083203.2* increases Cat Eye Syndrome Chromosome Region, Candidate 3 (*CECR3*) expression, impairing implantation (Feng *et al.,* 2018). Additionally, *NON-HSAT212577.1, NONHSAT035952.2, NONHSAT193031.1, NONHSAT053761.2,* and *NONHSAT025064.2* increase the expression of ST7 Overlapping Transcript 3 *(ST7-OT3),* DHRS4 Antisense RNA 1 *(DHRS4-AS1* ), chromosome 22 open reading frame 34 *(C22orf34),* RAMP2 Antisense RNA 1 *(RAMP2-AS1)* gene, and *PNCT_HSA157732,* respectively, in human stromal cells (Feng *et al.,* 2018).

Finally, the expression of *H19* decreases Insulin-like growth factor-binding protein 1 *(IGFBP1* ) expression in human endometrial tissue (Ariel *et al.,* 1997; Adriaenssens *et al.,* 1999; Kallen *et al.,* 2013). while *LINC473* decreases the expression of Prolactin (*PRL*), *IGFBP1,* Progesterone receptor (*PGR*), Forkhead box protein O1 (*FOXO1*), and Homeobox A10 (*HOXA10*) in human stromal cells (Chau *et al.,* 2002). *HOXA11* antisense expression in endometrial stromal cells decreases *HOXA11* expression and impairs implantation (Chau *et al.,* 2002). These genes help to improve implantation by increasing the proliferation in cell endometrium (Binart *et al.,* 2010).

In human serum, the expression of HIF-1alpha (*aHIF*) increases *VEGF* expression and impairs implantation (Qiu *et al.,* 2019), VEGF helps to improve implantation by increasing the rate of angiogenesis, and proliferation (Herington *et al.,* 2016) (Table 1).

### Potential Clinical Applications

The first successful IVF birth was reported in 1978 (Steptoe & Edwards, 1978), and since then, over eight million cases have been born through assisted reproductive methods, which have become popular worldwide (Edwards, 2005; Kamel, 2013). However, as previously stated, implantation failure can limit IVF therapy, which is mainly caused by insufficient endometrial receptivity. LncRNAs are important players in establishing endometrial receptivity, and their presence in serum is useful to diagnose receptivity and fertility (Feng *et al.,* 2018; Wang *et al.,* 2020b). Furthermore, as lncRNAs are being used as therapeutics in clinical trials in other fields (Bouckenheimer *et al.,* 2016; Feng *et al.,* 2018), their potential use in endometrial preparation in IVF is also being explored. The identification of LncRNAs can help in the development of diagnostic kits that may become predictive biomarkers for endometrial receptivity.

### LnCRNAs Improve Endometrial Receptivity

The majority of lncRNA therapeutic trials are today based on their inhibition of miRNAs or activation of genes (Chen, 2015). However, it is possible to register both as one patent. While most studies have focused on the therapeutic effect of lncRNAs on cancer, no drugs with lncRNA activating or inhibiting properties have been developed yet. Nevertheless, ongoing studies on the identification of lncRNAs related to infertility primarily target endometrial tissue. Currently, there are no lncRNA therapeutic drugs in clinical trials, and several technologies are involved in the transfer of lncRNA, with most being used to inhibit miRNAs. One of these techniques is nanotechnology and artificial exogenous extracellular vesicles (EVs), which facilitate the transfer of active or inhibiting lncRNA drugs to the target tissue (Wu *et al.,* 2021).

## CONCLUSIONS

Several research efforts have performed genome-wide analyses of differentially expressed lncRNAs in tissue, endometrial-derived cells, and women’s serum. Although differential expression of hundreds of lncRNAs has been reported, their roles are unclear. For example, 516 differentially expressed lncRNAs were reported by RNA sequencing assessment of human endometrial tissue derived from women with normal cycles during the secretory and proliferative stages, especially the implantation window (Sigurgeirsson *et al.,* 2017). Among the most expressed lncRNAs, nuclear-enriched abundant transcript 1 (*NEAT1*) can be mentioned (Sigurgeirsson *et al.,* 2017). However, the functional role of lncRNAs in the menstrual cycle cannot be understood only by the identification of differentially expressed lncRNA. Characterization assessments should be done to understand the functional and physiological roles of such lncRNAs in the endometrium.

Biomarkers capable of improving the embryo implantation success rate following IVF procedures should be identified and the prognosis and diagnosis of endometrial pathologies should also be considered. With increasing knowledge on the role and regulation of lncRNAs in the endometrium deepens, we can expect to gain a better understanding of the normal and abnormal physiology of the endometrium, resulting in novel diagnostics and therapies according to the biological feature of such regulatory RNAs.
